# Zinc Supplementation Partially Decreases the Harmful Effects of a Cafeteria Diet in Rats but Does Not Prevent Intestinal Dysbiosis

**DOI:** 10.3390/nu14193921

**Published:** 2022-09-22

**Authors:** Samia Squizani, Jeferson Jantsch, Fernanda da Silva Rodrigues, Matheus Filipe Braga, Sarah Eller, Tiago Franco de Oliveira, Alexandre Kleber Silveira, José Cláudio Fonseca Moreira, Marcia Giovenardi, Marilene Porawski, Renata Padilha Guedes

**Affiliations:** 1 Programa de Pós-Graduação em Biociências, Universidade Federal de Ciências da Saúde de Porto Alegre (UFCSPA), Porto Alegre 90050-170, Brazil; 2 Acadêmico do Curso de Biomedicina, Universidade Federal de Ciências da Saúde de Porto Alegre (UFCSPA), Porto Alegre 90050-170, Brazil; 3 Programa de Pós-Graduação em Ciências da Saúde, Universidade Federal de Ciências da Saúde de Porto Alegre (UFCSPA), Porto Alegre 90050-170, Brazil; 4 Departamento de Bioquímica, Universidade Federal do Rio Grande do Sul (UFRGS), Porto Alegre 90035-003, Brazil; 5 Programa de Pós-Graduação em Medicina: Hepatologia, Universidade Federal de Ciências da Saúde de Porto Alegre (UFCSPA), Porto Alegre 90050-170, Brazil

**Keywords:** obesity, cafeteria diet (CAF), inflammation, gut microbiota, short-chain fatty acid (SCFA), zinc (Zn)

## Abstract

Zinc (Zn) plays an important role in metabolic homeostasis and may modulate neurological impairment related to obesity. The present study aimed to evaluate the effect of Zn supplementation on the intestinal microbiota, fatty acid profile, and neurofunctional parameters in obese male Wistar rats. Rats were fed a cafeteria diet (CAF), composed of ultra-processed and highly caloric and palatable foods, for 20 weeks to induce obesity. From week 16, Zn supplementation was started (10 mg/kg/day). At the end of the experiment, we evaluated the colon morphology, composition of gut microbiota, intestinal fatty acids, integrity of the intestinal barrier and blood–brain barrier (BBB), and neuroplasticity markers in the cerebral cortex and hippocampus. Obese rats showed dysbiosis, morphological changes, short-chain fatty acid (SCFA) reduction, and increased saturated fatty acids in the colon. BBB may also be compromised in CAF-fed animals, as claudin-5 expression is reduced in the cerebral cortex. In addition, synaptophysin was decreased in the hippocampus, which may affect synaptic function. Our findings showed that Zn could not protect obese animals from intestinal dysbiosis. However, an increase in acetate levels was observed, which suggests a partial beneficial effect of Zn. Thus, Zn supplementation may not be sufficient to protect from obesity-related dysfunctions.

## 1. Introduction

Zinc (Zn) is a mineral widely distributed throughout the human body in small concentrations, and is involved in several biochemical and enzymatic processes [1,2,3]. It is an enzymatic cofactor for several enzymes that regulate the metabolism of carbohydrates, proteins, and lipids [1,3]. In addition, it exerts beneficial effects on immune function and inflammatory response, and contributes to barrier integrity and cognitive processing [2,3,4,5,6,7]. Moreover, it has already been established that obesity can affect the distribution of Zn in the body, reducing its systemic availability in obese individuals [8,9]. Previous studies have shown that Zn supplementation may improve anthropometric measurements and reduce inflammatory markers, insulin resistance, oxidative stress, and obesity-related neuroinflammation. Thus, Zn supplementation reduces metabolic dysfunction and may decrease neurological impairment related to obesity [10,11,12]. 

The consumption of processed energy-dense food, combined with overeating, urbanization, and a sedentary lifestyle in modern Western societies, is likely a major contributor to the obesity epidemic [13,14,15]. Obesity is associated with low-grade inflammation characterized by an increased production of proinflammatory cytokines, such as tumor necrosis factor-α (TNF-α), interleukin-1β (IL-1β), and interleukin-6 (IL-6) [16,17,18]. As a result of this inflammatory state, this complex and multifactorial disease is implicated in a higher prevalence of metabolic disorders, such as type 2 diabetes, cardio-metabolic disease, non-alcoholic liver disease, and also dysbiosis of the gut microbiota and neurological impairment [15,19].

Another consequence of low-grade systemic inflammation is the disruption of the intestinal epithelial barrier and blood–brain barrier (BBB), which leads to neuroinflammation [20,21]. The BBB comprises a tight junction complex and is assembled by several proteins, such as transmembrane, cytoplasmic attachment, and cytoskeleton proteins that form epithelial barriers [22,23]. Zonula occludens-1 (ZO-1), claudin-5, and occludin are recognized as markers of the integrity of the BBB due to their critical role in maintaining the integrity of tight junctions [24,25].

In addition to BBB dysfunction and neuroinflammation, neuroplasticity might be affected following inflammatory conditions, such as obesity. Brain-derived neurotrophic factor (BDNF) is a protein of the neurotrophin family that is essential for neuronal development, maintenance, survival, cognitive function, and synaptic plasticity. In addition, it is considered a key regulator of synaptic transmission, mainly in the hippocampus and neocortex [6,26]. Synaptophysin is a protein present in presynaptic vesicles. It participates in synaptic formation and neurotransmitter release. Additionally, it is used as a marker of synapsis distribution and density [27,28,29]. Thus, in the present study, we investigated BDNF and synaptophysin to assess whether consuming ultra-processed and hypercaloric food might affect neuroplasticity.

Regarding gut microbiota, there is a high variability of microorganisms in their composition depending on dietary habits [30,31]. Additionally, several diseases can be associated with a certain microbiota profile [32,33,34]. Previous studies have indicated that obesity is linked to disruptions in the gut microbiota composition, creating an imbalance in the microbial ecosystem defined as dysbiosis [35,36,37]. In addition, in healthy conditions, commensal anaerobic colonic bacteria produce functional metabolites by highly fermentable dietary fibers and resistant starch that benefit their host [38]. Short-chain fatty acids (SCFA) are functional metabolites represented mainly by acetate, butyrate, and propionate. These metabolites promote intestinal epithelium integrity, regulate immune function, modulate neurotransmission, and appear involved in neuroimmunoendocrine regulation [39,40]. Unbalanced SCFA concentrations due to consuming a Western diet can lead to dysbiosis. Subsequently, this leads to an increased intestinal permeability and induces low-grade systemic inflammation [19,41].

Although Zn’s role in decreasing inflammation and improving cognition is already described, its effect on reversing the harmful consequences of consuming ultra-processed foods on microbiota composition and neuroplasticity is still unclear. The main aim of this study was to investigate the effects of Zn supplementation on the intestinal microbiota, intestinal barrier, and BBB in Wistar rats fed with a cafeteria diet (CAF). The CAF mimics the foods consumed by Western civilization by exposing the animals to highly palatable energy-dense foods with a high content of saturated fat and refined sugars and a large amount of food additives [42,43]. This model elicits obesity-related disorders, such as dysbiosis, leaky gut, metabolic disorders, low-grade systemic inflammation, neuroinflammation, and behavioral dysfunction in rodents [42,44]. As expected, the chronic consumption of the CAF for 20 weeks increased body weight, as shown in a previous study [10]. Thus, the present work also aimed to evaluate Zn’s ability to reverse the mentioned harmful effects of obesity, since its supplementation started after obesity had already been developed.

## 2. Materials and Methods

### 2.1. Animals

Male three-month-old Wistar rats were obtained from the facility of the Federal University of Health Sciences of Porto Alegre (UFCSPA). Only male rats were used to avoid hormonal fluctuation and a possible impact on the results of the study. During the period of diet administration, one or two animals were housed per cage in a temperature-controlled environment (21 °C ± 2 °C) under a 12 h light/dark cycle and with ad libitum diet and water. The study was approved by the Institutional Animal Care and Use Committee of UFCSPA under the protocol number 570/18. All research procedures were designed to minimize the number of animals and suffering.

### 2.2. Experimental Groups and Diet

Twenty-eight animals were divided into four groups (*n* = 7/group), namely: (i) the control group (CT); (ii) control group + zinc (CT Zn); (iii) the cafeteria group (CAF), and (iv) the cafeteria + zinc group (CAF Zn). Standard chow (CT) or cafeteria (CAF, high fat and high calorie) diets were administered for 20 weeks. The cafeteria diet consisted of the alternating offer of three menus per week, containing bacon mortadella (Perdigão^®^, Itajaí, Brazil), strawberry-flavored biscuits (Isabela^®^, Bento Gonçalves, Brazil), chocolate biscuits (Isabela^®^, Bento Gonçalves, Brazil), pizza-flavored hot crackers (Parati^®^, São Lourenço do Oeste, Brazil), white chocolate (Harald^®^, Santana de Parnaíba, Brazil), orange-flavored soda (Sukita^®^, Sapucaia do Sul, Brazil), sausages (Alibem^®^, Porto Alegre, Brazil) offered concomitantly with ad libitum standard chow, and water. The alternation between cafeteria diet menus was performed every two days in order to maintain novelty. This diet’s average energy supply was 4.5 Kcal/g (42% carbohydrates, 16% proteins, 42% lipids). The standard diet consisted of the standard chow Nuvilab CR-1 provided by the animal facility of UFCSPA (Nuvital^®^, Colombo, Brazil), which had an average energy supply of 3.4 Kcal/g (63% carbohydrates, 26% proteins, 11% lipids). The total energy content of the CAF was calculated based on manufacturer’s information. Leftovers were evaluated, and food consumption was calculated at the end of the experiment. The average energy consumption of CT groups was 771.5 Kcal/day, while CAF groups consumed 963 Kcal/day [10].

### 2.3. Zn Treatment

Chelated zinc bisglycinate (Deconto Farma^®^, Porto Alegre, Brazil) was administered at a dose of 10 mg/Kg/day by gavage for 4 weeks from the 16th week of the diets. Gavage was performed daily between 2 and 3 pm, during the light cycle. Animals that did not receive zinc supplementation were also subjected to gavage with vehicle solution (water, 0.5 mL per animal). Animals were weighed weekly to determine weight gain. It should be noted that the treatment with zinc was started after obesity had already been induced. Zn content in the liver and cerebellum was determined by flame atomic absorption spectrometry (model AA 7000F; Shimadzu, Kyoto, Japan) equipped with a hollow cathode lamp and a deuterium lamp as a background corrector, and dosage was previously published [10].

### 2.4. Tissue, Blood and Feces Collection

At the end of the experimental period of 20 weeks, the animals were euthanized by decapitation without anesthesia. After that, feces were collected directly from the proximal colon. The proximal portion of the colon and brain were also collected. The brain was dissected out and the hippocampus and cerebral cortex were stored at −80 °C for further homogenization and analysis.

### 2.5. Histological Analysis 

Histology of the proximal colon was performed for the general morphological analysis. Three to five animals per group were used for histology. For this, the tissue was fixed in paraformaldehyde, posteriorly embedded in a paraffin block, and cut transversely to a thickness of 4 μm. After this, the sections were stained with hematoxylin and eosin (HE). The slides were then analyzed under an EVOS microscope (EVOS Cell Imaging Systems, Thermo Fisher Scientific, Waltham, MA, USA). At least six measurements were taken per animal at 10× magnification. The crypt depth was measured using ImageJ software, and the size was normalized to a 500 µm scale [45].

### 2.6. Next-Generation Sequencing 16s rRNA

Stool samples were collected in sterile tubes and immediately stored at −20 °C. We also used samples from CT and CAF groups provided from a previous experiment using the same diet protocol, and the sample size was 8 animals for these groups. The genomic material of microbial DNA was obtained from approximately 200 mg of a fecal sample with a specific extraction kit (Microbiome DNA Purification kit, Invitrogen^®^, Waltham, MA, USA) following the manufacturer’s instructions. After DNA extraction, the quantification of the DNA of each sample was performed by a NanoDrop spectrophotometer (Shimadzu, Japan). The libraries were prepared from an average of 5 to 10 μg of genomic material.

The hypervariable V3-V4 region from the 16S ribosomal RNA (rRNA) gene was amplified through PCR using genomic DNA (approximately 50 ng per reaction) and the following primer pair: 515F (5′-GTGCCAGCMGCCGCGGTAA-3′) and 806R (5′-GGACTACHVGGGTWTCTAAT-3′). In order to pool different samples in the same reaction, the primer-fusion method and each sample had a distinct barcode attached to the corresponding PCR product. The amplification was performed using Platinum™ PCR SuperMix High Fidelity (Invitrogen^®^, Waltham, MA, USA). The products were verified through electrophoresis in an agarose gel, purified with the AMPure XP PCR Purification Kit (Cat# A63881, Beckman Coulter Inc. Life Sciences, Indianapolis, IN, USA), quantified using Qubit™ dsDNA HS Assay Kit (Invitrogen^®^, Waltham, MA, USA) and subjected to emulsion PCR using the Ion PGM™ Hi-Q™ View OT2 Kit (Cat# A29900, Thermo Fisher Scientific, Waltham, MA, USA). Afterwards, the resulting enriched beads were sequenced in a next-generation sequencing (NGS) machine (Ion Torrent PGM™, Life Technologies, Carlsbad, CA, USA) using the Ion PGM^™^ Hi-Q^™^ View Sequencing Kit (Cat# A30044, Thermo Fisher Scientific, Waltham, MA, USA).

The 16S rRNA reads generated by high-throughput sequencing were submitted to a quality control analysis that retained sequences with a minimum length of 100 base pairs and trimmed the sequences to remove low-quality bases to obtain a minimum Phred score of 30 using PRINSEQ [46]. The remaining sequences were dereplicated and sorted by decreasing read abundance and filtered to exclude singletons using USEARCH v7.0.1090. Clusters were assembled using a minimum identity of 99% and chimeras were removed using the Ribosomal Database Project (RDP) reference database [47].

The taxonomic assignment was obtained using QIIME v1.7 [48]. Operational taxonomical units (OTUs) were selected based on 97% sequence similarity and taxonomic data were obtained using a classification algorithm with the 97% OTUs version of GreenGenes 13.8 [49]. Diversity index was calculated utilizing the vegan:ecological diversity package in R.

### 2.7. Determination of Fatty Acid Profile

The levels of saturated fatty acid (SFA) and SCFA were measured in the large intestine of the animals. The determination of SFA was performed by gas chromatography coupled to mass spectrometry (GC-MS). For this, 100 mg of intestinal tissue samples were homogenized in 1 mL of deionized water. After that, an aliquot of 50 µL of the homogenate was used for the extraction of the fatty acids with the addition of 950 µL of a chloroform/methanol (2:1, *v*/*v*) solution and one drop of concentrated hydrochloric acid (HCl) was added to a plastic tubes, forming a single liquid phase. Subsequently, 200 µL of deionized water was added, promoting a separation between the chloroform phase (rich in lipids) and the methanol/aqueous phase. The samples were decanted for 5 min at room temperature. Then, the entire fraction containing chloroform was collected, dried under nitrogen flow at room temperature, and stored at −20 °C. Thereafter, the samples were finally resuspended in 30 µL of methanol and 2 µL aliquots and then injected into an analytical system consisting of a gas chromatograph model GC-2010 A PLUS coupled to the QP-2010 Ultra mass spectrometer (Shimadzu, Japan) adapted from G. DEMERS et al. [50]. The spectrometer was operated in scan mode (50–700 *m*/*z*) and the results obtained were verified by comparing the findings with those described in the mass spectral reference library of the National Institute of Standards and Technology (NIST).

The determination of SCFA in the large intestine was performed by liquid chromatography with tandem mass spectrometry (LC-MS/MS), according to a protocol developed in-house. For this, 50 μL of the homogenate, 20 μL NaOH (2 M) and 175 μL of HCl (2 M) were added to plastic tubes followed by vortexing for 30 s. The samples were then centrifuged for 6 min at 12,000× *g*. After that, an aliquot of 175 µL of the supernatant was collected and transferred to a glass vial. In order to promote fatty acid derivatization, 25 μL of 2,4-Dinitrophenylhydrazine (DNPH) was added. The flask was closed and incubated for 30 min at 40 °C. Then, 100 μL of the mixture was transferred to a vial and 100 μL of acetonitrile was added. Thereafter, an aliquot of 10 μL was injected into the analytical system equipment. The LC-MS/MS equipment consisted of a chromatographic system Nexera UFLC (Shimadzu, Japan) equipped with two binary pumps (LC-30AD), a column oven (CTO-30A), a diode array absorbance detector (SPD-M20A), and an automatic injector (SIL-30AMP) coupled with a quadrupole mass spectrometer model LCMS-8045 (Shimadzu, Japan). The results obtained were processed and evaluated using LabSolutions software (Shimadzu, Japan).

### 2.8. Western Blotting

The protein expression of ZO-1, claudin-5, BDNF, and synaptophysin was analyzed in the cerebral cortex, hippocampus and proximal colon. Tissues were processed and homogenized in a lysis buffer, and then samples were centrifuged for 10 min at 8000 rpm. After the protein quantification by Bradford protein assay, Laemmli buffer was mixed with 30 µg of proteins and heated at 90 °C for 2 min. Proteins were loaded and separated by SDS-PAGE (sodium dodecyl sulfate polyacrylamide gel electrophoresis) gel and later transferred to nitrocellulose membranes using a transfer system with semi-dry equipment (mini Trans-blot Electrophoretic Transfer Cell, BioRad, Hercules, CA, USA) at 110 V for 1–2 h. Membranes were incubated with 8% powdered milk in saline Tris buffer containing 0.1% tween 20 (T-TBS) for 90 min in order to block nonspecific binding. Membranes were incubated overnight (at 4 °C) with the primary antibody. Primary antibodies to TLR-4 (1:500, Cat# sc-293072, Santa Cruz Biotechnology^®^, Dallas, TX, USA), ZO-1 (1:500, Cat# 61-7300, Invitrogen^®^, Waltham, MA, USA), claudin-5 (1:1000, Cat# ABT45, Merk^®^, Kenilworth, NJ, USA), BDNF (1:1000, Cat# BS-4989R, Thermo Fisher Scientific, Waltham, MA, USA), and synaptophysin (1:500, Cat# MA5-14532, Thermo Fisher Scientific, Waltham, MA, USA) were used. Then, the membranes were incubated with secondary anti-mouse (Cat# A9044, Sigma-Aldrich, St. Louis, MI, USA) or anti-rabbit (Cat# AP132P, Merk, Kenilworth, NJ, USA) antibodies for 2 h at room temperature. All incubations were performed under constant agitation, and between each incubation membranes were washed with T-TBS. A chemiluminescence reaction was performed to detect the labeled proteins and the images were obtained using the ChemiDoc MP photodocumenter (Bio-rad Laboratories, Hercules, CA, USA). The results of each membrane were relative to the values found by incubating them with the primary antibody anti-β-actin (1:500, Cat# sc-47778 horseradish peroxidase, Santa Cruz Biotechnology^®^, Dallas, TX, USA) or with nonspecific bands [51]. To avoid inter-assay variations, samples from all experimental groups were processed in parallel.

### 2.9. Statistical Analysis

Data are expressed as the mean and standard error of the mean (SEM). Two-way analysis of variance (ANOVA) was performed. The main factors were diet (CT or CAF) and supplementation (vehicle or zinc). The Bonferroni test was used for post hoc analysis. The value of *p* < 0.05 was considered as indicative of statistical significance. All analyses were performed using the GraphPad Prism^®^ 9.0 program (San Diego, CA, USA).

## 3. Results

### 3.1. Colon Morphology

**Figure 1:**
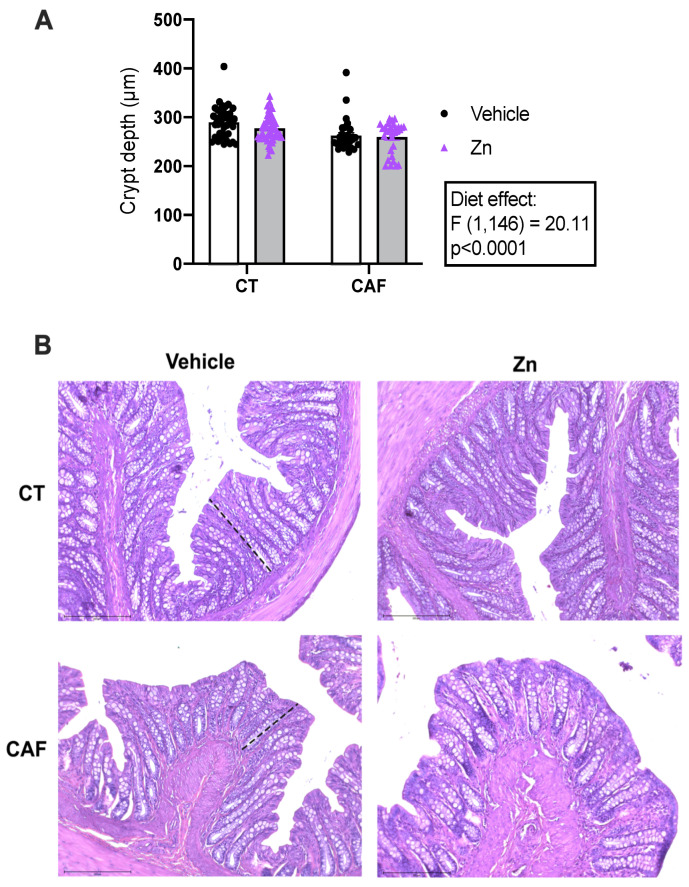
Crypt depth measurement in the proximal portion of the colon of control (CT) and cafeteria diet (CAF)-fed rats. (**A**) Crypt depth was reduced following CAF. (**B**) Representative images of each group of the colon stained with hematoxylin and eosin. The dashed lines indicate crypt length. Scale bar: 200 μm. The text box indicates significant differences shown by two-way ANOVA regarding the effects of the diet (CT and CT + Zn vs. CAF and CAF + Zn) and Zn treatment (CT and CAF vs. CT + Zn and CAF + Zn). *n* = 3–5 animals/group, 10 measurements per animal.

The analysis of the crypt depth of the colon is shown in Figure 1. Crypt depth was lower in CAF-fed animals when compared with CT-fed animals (diet effect: F1,146 = 20.11, *p* < 0.0001). Zn supplementation did not exert any effect on crypt depth measurements.

### 3.2. Composition of the Gut Microbiota

**Figure 2:**
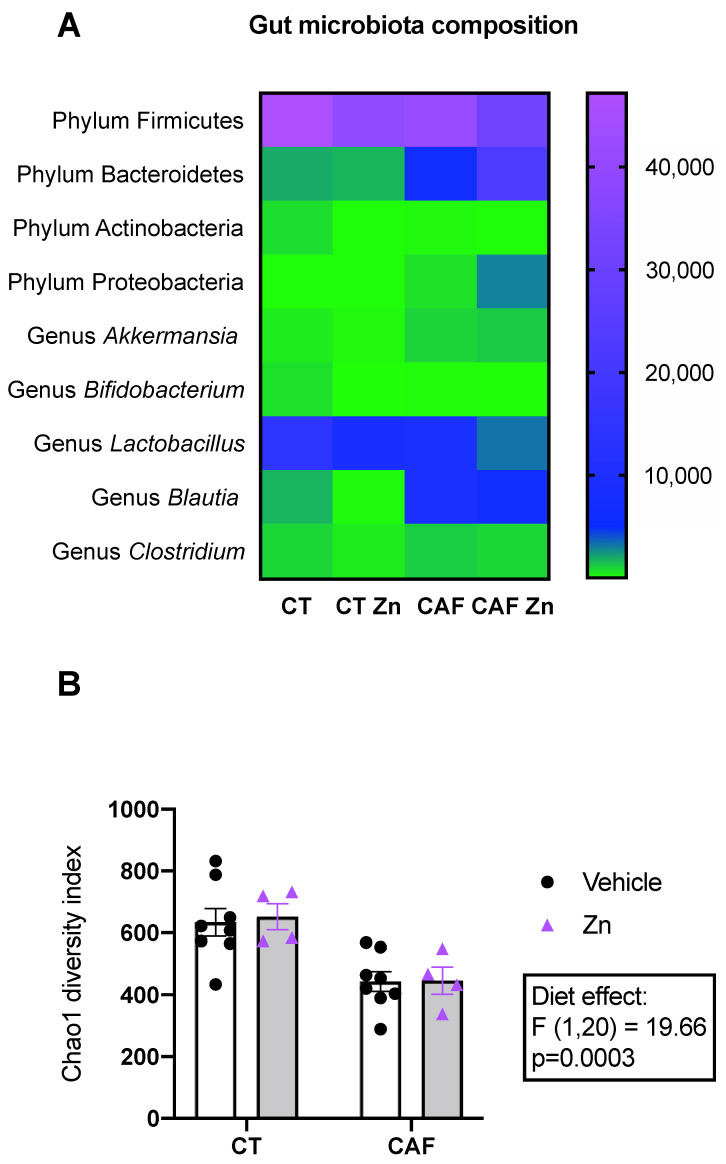
Composition of the gut microbiota of control (CT) and cafeteria diet (CAF)-fed animals. (**A**) Heatmap shows the phylum and genera levels of the main bacterial community found in the fecal samples of the intestinal colon. The range of colors, from green to lilac, indicates the abundance of each phylum or genera per group. (**B**) Chao1 diversity index. The text box indicates significant differences shown by two-way ANOVA regarding the effects of the diet (CT and CT + Zn vs. CAF and CAF + Zn). *n* = 4–8 animals/group.

We performed the next-generation sequencing of the V4 16S rRNA region for the taxonomic identification of intestinal bacterial composition. The heatmap (Figure 2A) shows the number of reads of the main phyla and genera. Significant differences regarding diet and Zn supplementation (two-way ANOVA main effects) and the multiple comparisons (post hoc Bonferroni test) are described below.

We found a decrease in the Firmicutes phylum in CAF-fed rats (diet effect, F1,9 = 13.55, *p* = 0.0016). Additionally, the post hoc test showed a significant decrease in Firmicutes bacteria in CAF + Zn compared to the CAF group (*p* = 0.0307). On the other hand, Bacteroidetes phylum was significantly increased following CAF (diet effect, F1,16 = 40.83, *p* < 0.0001), but it also showed a Zn supplementation effect (F1,16 = 5.596, *p* = 0.0310) and an interaction between diet and Zn (F1,16 = 7.121, *p* = 0.0168). The Bonferroni post hoc test also showed an increase in CAF + Zn compared to the CAF group (*p* = 0.0079). These findings show that Zn supplementation was able to decrease the abundance of Firmicutes bacteria while increasing Bacteroidetes phylum in CAF + Zn group. There was no difference in the abundance of Actinobacteria phylum among the groups. The Proteobacteria phylum showed an interaction between diet and Zn (F1,17 = 5.991, *p* = 0.0255), a diet effect (F1,17 = 65.03, *p* < 0.0001), and a Zn effect (F1,17 = 5.838, *p* = 0.0272). Additionally, CAF + Zn showed a significantly higher abundance of Proteobacteria when compared to the CAF group (*p* = 0.0101). These findings show, once again, that Zn supplementation was able to increase the abundance of Proteobacteria phylum only in CAF-fed rats. 

Regarding the genus taxonomic level, we analyzed the *Akkermansia*, *Bifidobacterium*, *Blautia*, *Clostridium* and *Lactobacillus*. There was an increase in the abundance of *Akkermansia* (diet effect, F1, 18 = 6.520, *p* = 0.0200) and *Blautia* (diet effect, F1,18 = 36.07, *p* < 0.0001) in CAF-fed rats, with no effect of Zn supplementation for either genus. Concerning *Lactobacillus*, we found an interaction between diet and Zn (F1,17 = 6.583, *p* = 0.0201), diet effect (F1,17 = 37.34, *p* < 0.0001) and Zn effect (F1,17 = 13.80, *p* = 0.0017). A significant decrease was also observed in *Lactobacillus* abundance in CT + Zn compared to the CT group (*p* = 0.0008). These findings show that Zn supplementation was able to reduce the abundance of *Lactobacillus* only in lean rats. There was no difference in the abundance of *Bifidobacterium* and *Clostridium* genera among the groups.

The diversity of the gut microbiota based on the Chao1 index (Figure 2B) was also analyzed, which showed a reduction in the CAF-fed groups (diet effect, F1,20 = 19.66, *p* = 0.0003) with no effect of Zn supplementation.

### 3.3. SCFA, MCFA, LCFA and VLCFA Profile in the Intestine

**Figure 3:**
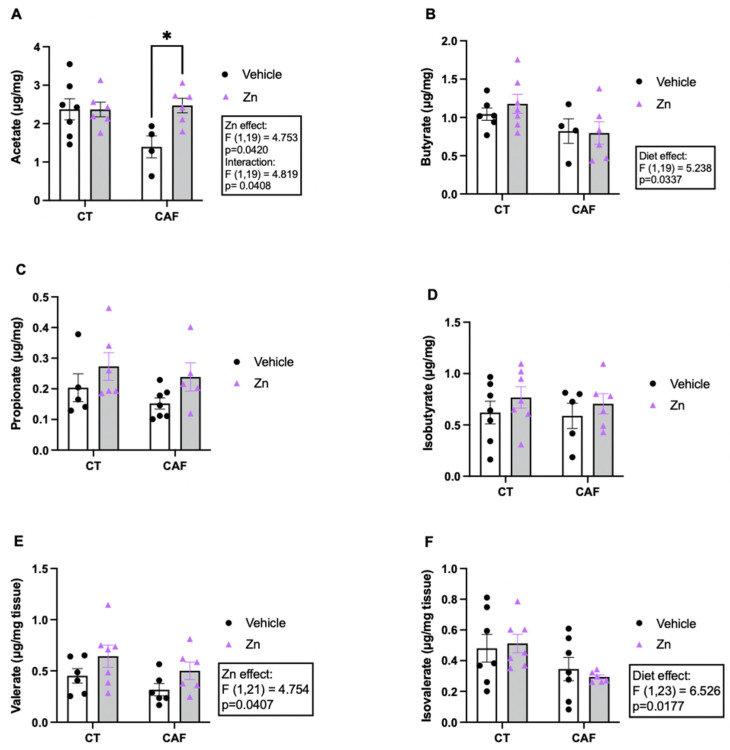
Short-chain fatty acid (SCFA) concentration in the proximal portion of the colon of control (CT) and cafeteria diet (CAF)-fed rats. (**A**) Acetate, (**B**) butyrate, (**C**) propionate, (**D**) isobutyrate, (**E**) valerate, and (**F**) isovalerate levels. The text box indicates significant differences shown by two-way ANOVA regarding the effects of diet (CT and CT + Zn vs. CAF and CAF + Zn), Zn supplementation (CT and CAF vs. CT + Zn and CAF + Zn), and the interaction between diet and Zn supplementation. Multiple comparisons were performed by Bonferroni post hoc test and significant differences are shown by the asterisk (* *p* < 0.05). *n* = 4–7 animals/group.

The determination of SCFA in the intestine is shown in Figure 3. The acetate concentration (Figure 3A) showed an interaction between diet and Zn (F1,19 = 4.819, *p* = 0.0408) and a Zn supplementation effect (F1,19 = 4.753, *p* = 0.0420). In the post hoc comparisons, an increase in acetate levels was observed in the CAF + Zn group compared to CAF group (*p* = 0.0188). These findings demonstrate that Zn supplementation in obese rats could return the acetate concentration to the level of the lean animals. However, there was a diet effect (F1,19 = 5.238, *p* = 0.0337) in butyrate levels (Figure 3B) which was decreased in CAF-fed rats with no effect of Zn supplementation. We noticed no difference in the concentration of propionate (Figure 3C) and isobutyrate (Figure 3D) among the groups. The valerate concentration (Figure 3E) increased in Zn-supplemented rats (Zn effect, F1,21 = 4.754, *p* = 0.0407), with no effect of diet. Furthermore, we found a decreased concentration of isovalerate (Figure 3F) (diet effect, F1,23 = 6.526, *p* = 0.0177) in CAF-fed rats, with no effect of Zn supplementation. 

The determination of medium-chain fatty acid (MCFA), long-chain fatty acid (LCFA) and very long-chain fatty acid (VLCFA) concentration in the intestinal tissue (µg/mg) is shown in Table 1. Regarding the MCFAs, we analyzed the concentration of caprylic, decanoic, octanoic, lauric, and undecanoic acids. There was an increase in decanoic (diet effect, F1,23 = 8.969, *p* = 0.0065), octanoic (diet effect, F1,23 = 5.175, *p* = 0.0326) and lauric (diet effect, F1,23 = 11.49, *p* = 0.0025) fatty acids in CAF-fed rats, with no effect of Zn supplementation. The undecanoic fatty acid showed an interaction effect between diet and Zn (F1,23 = 5.716, *p* = 0.0254); in this case, post hoc analysis showed that the CT + Zn group was higher than the CT group (*p* = 0.0285). Additionally, we observed a decreased concentration of caprylic acid (diet effect, F1,22 = 4.387, *p* = 0.0479) in CAF-fed rats, with no effect of Zn supplementation. Moreover, we determined the concentration of the LCFAs such as elaidic, heptadecanoic, linoleic, myristic, myristoleic, palmitic, pentadecanoic, stearic, and tridecanoic. Similarly to the findings of the MCFAs, we noticed an increase in the concentration of elaidic (diet effect, F1,23 = 34.12, *p* < 0.0001), linoleic (diet effect, F1,23 = 14.46, *p* = 0.0009), myristic (diet effect, F1,23 = 32.76, *p* < 0.0001), palmitic (diet effect, F1,19 = 10.15, *p* = 0.0049), and stearic (diet effect, F1,21 = 6.600, *p* = 0.0179) acids in response to CAF, with no effect of Zn. Additionally, the myristoleic acid showed an interaction effect between diet and Zn (F1,20 = 6.217, *p* = 0.0215). There was no difference in the concentration of heptadecanoic, pentadecanoic, and tridecanoic acids among the groups. As for VLCFA, we analyzed the concentration of behenic, heneicosanoic, lignoceric, and tricosanoic acids. Again, an increase in behenic (diet effect, F1,23 = 26.61, *p* < 0.0001) and heneicosanoic (diet effect, F1,23 = 13.62, *p* = 0.0012) acids was observed in CAF-fed animals, with no effect of Zn supplementation. No differences were seen in the concentration of lignoceric and tricosanoic acids among the groups.

### 3.4. Blood–Brain Barrier (BBB) and Intestinal Barrier Integrity Components

**Figure 4:**
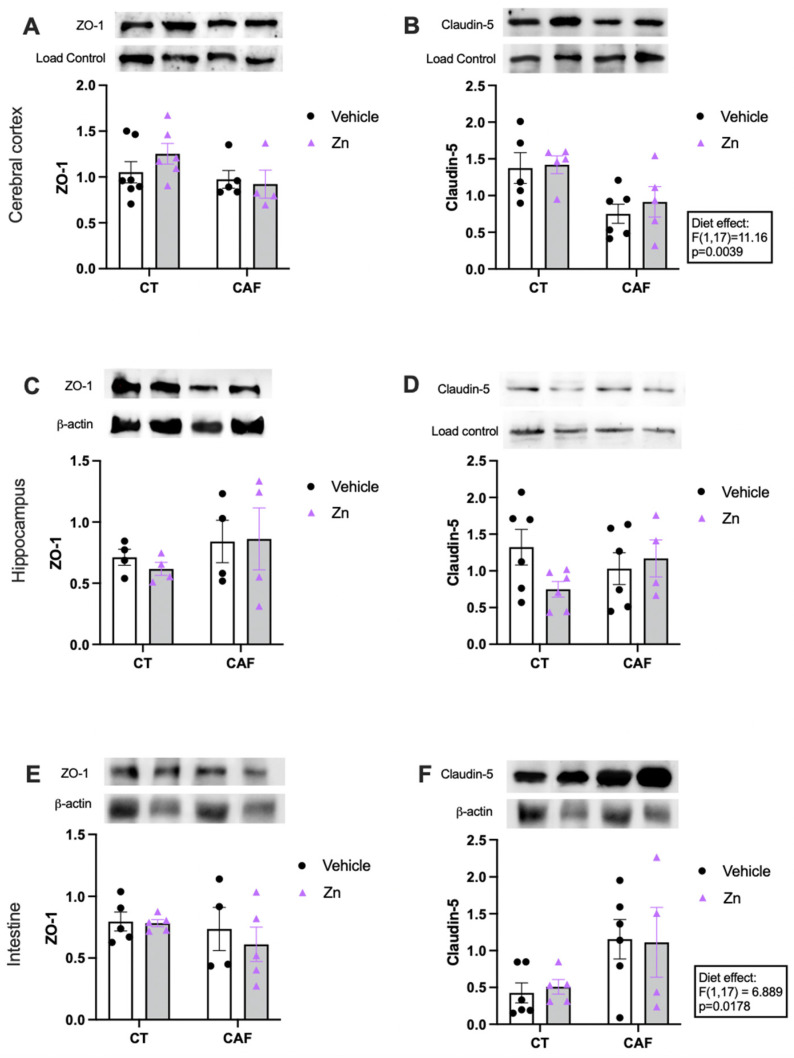
Protein expression of zonula occludens 1 (ZO-1) and claudin-5 in cerebral cortex (**A**,**B**), hippocampus (**C**,**D**), and proximal portion of the intestinal colon (**E**,**F**) in control (CT) and cafeteria diet (CAF)-fed rats. Representative bands of each group are shown on the top of the graphs. β-actin or nonspecific bands were used as a loading control. The text box indicates significant differences shown by two-way ANOVA regarding effects of the diet (CT and CT + Zn vs. CAF and CAF + Zn). *n* = 4–7 animals/group.

The protein expressions of ZO-1 and claudin-5 in the cerebral cortex, hippocampus, and proximal portion of the intestinal colon are shown in Figure 4. There was no difference in the ZO-1 expression in the cerebral cortex (Figure 4A), hippocampus (Figure 4C), or intestinal colon (Figure 4E) among the groups. However, there was a decrease in claudin-5 protein expression in the cerebral cortex (Figure 4B) of CAF-fed animals (diet effect, F1,17 = 11.16, *p* = 0.0039), with no effect of Zn supplementation. Intriguingly, we also observed a diet effect (F1,17 = 6.889, *p* = 0.0178) in claudin-5 expression in the intestinal colon (Figure 4F) which was increased in CAF-fed rats, with no effect of Zn supplementation. Claudin-5 did not change among groups in the hippocampus (Figure 4D).

### 3.5. Synaptic and Neuroplasticity Markers

**Figure 5:**
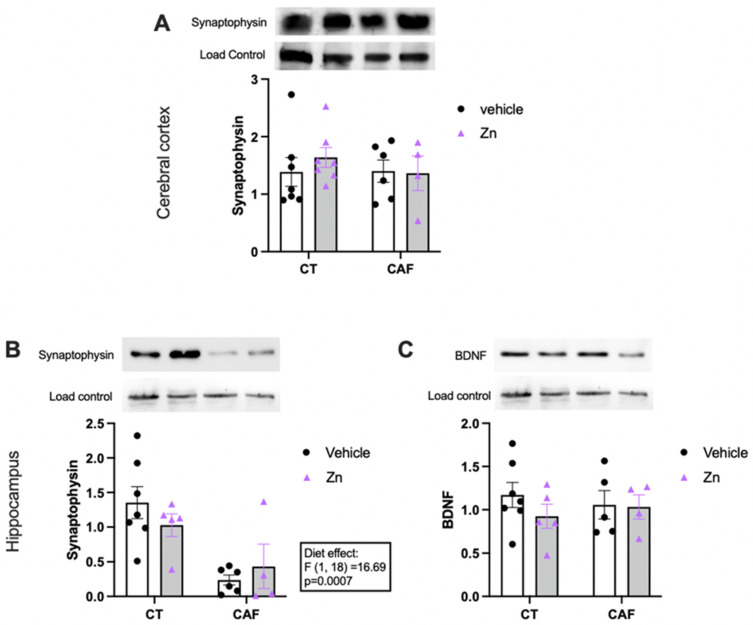
Protein expression of synaptophysin and brain-derived neurotrophic factor (BDNF) in the cerebral cortex (**A**) and hippocampus (**B**,**C**) of control (CT) and cafeteria diet (CAF)-fed rats. Representative bands of each group are shown on the top of the graphs. β-actin or nonspecific bands were used as a loading control. The text box indicates significant differences shown by two-way ANOVA regarding the effects of diet (CT and CT + Zn vs. CAF and CAF + Zn). *n* = 4–7 animals/group.

The protein expressions of synaptophysin and brain-derived neurotrophic factor (BDNF) in the cerebral cortex and hippocampus are shown in Figure 5. We found no difference in the protein expression of synaptophysin (Figure 5A) in the cerebral cortex among the groups. However, there was a lower expression of synaptophysin in the hippocampus (Figure 5B) of CAF-fed animals (diet effect, F1,18 = 16.69, *p* = 0.0007), with no effect of Zn supplementation. Moreover, BDNF expression in the hippocampus (Figure 5C) did not change among the groups.

## 4. Discussion

We have previously shown that Zn supplementation could decrease metabolic dysfunction and neuroinflammation and improve memory in obese rats fed with a CAF [10]. Here, we showed that Zn changed the microbiota composition but did not enhance its diversity in obese animals. Additionally, Zn was able to increase acetate levels in obese rats. On the other hand, Zn did not affect the intestinal or cerebral barriers’ integrity or the expression of neuroplasticity markers, such as synaptophysin and BDNF. Thus, we show that when obesity is severe, as in the CAF experimental model, the effects of Zn supplementation may not be as beneficial as in other populations, such as normal body weight or even overweight people. These findings might help to delimit the recommendation of Zn supplementation to these groups.

There are some controversial findings regarding intestinal morphology in diet-induced obesity (DIO) models. Some studies reported an increase in the crypt depth or villi height in obesity [52,53,54]. Zhou and collaborators found this result in the duodenum and jejunum but found no differences in the colons of mice [55]. It was also demonstrated that titanium dioxide, a coloring food additive, alters the functional absorption of nutrients by decreasing the number of microvilli and, consequently, reducing absorptive area. In addition, the same study showed that titanium dioxide significantly decreased zinc transport [56]. Our findings showed that chronic ingestion of the CAF exerts a decrease in the crypt depth of the colon in adult rats, with no effect of Zn supplementation. These findings may be related to the food additives present in the CAF.

In the present study, we also found that the CAF modulated the intestinal microbiota composition and induced changes on its profile. Although there is a lack of definition of a “healthy microbiota” due to the high variability of microorganisms among the microbiota composition, there are some patterns at taxonomic levels of phyla in all vertebrates [57,58]. In fact, several diseases are associated with a certain microbiota profile [32,33,34].

We demonstrated that CAF induced a decrease in Firmicutes and an increase in Bacteroidetes phyla abundance. In obese rats, Zn decreased Firmicutes while increasing Bacteroidetes. There is some divergence among studies regarding the abundance of these phyla. Although some reports demonstrated an increase in Firmicutes and a decrease in Bacteroidetes in obesity [42,59,60], others showed the opposite, or no difference in either phylum [44,61]. However, the divergence in the literature may be related to different diet protocols. It has already been shown that diet composition, including macronutrient and micronutrient distribution, or even the presence of food additives, can modulate the gut microbiota profile [42,62,63]. Furthermore, non-nutritive sweeteners, including acesulfame potassium, saccharin, and sucralose, exert a strong bacteriostatic effect. The consumption of these sweeteners can selectively inhibit the survival taxa of some bacterial populations, consequently changing gut microbiota homeostasis [64]. Since CAF consists of several ultra-processed foods and some of them have in their composition these sweeteners, it could have influenced our findings. In addition, the increase in the abundance of Bacteroidetes by Zn supplementation in CAF-fed rats seems interesting since it consists of several bacteria involved with SCFA production [30]. Additionally, the higher proportion of Firmicutes phyla in obese rodents fed with a Western diet may be associated with an increase in the abundance of *Clostridium ramosum*, which has been linked to metabolic syndrome [65]. Therefore, based on these findings, a protective effect of Zn supplementation could be suggested in CAF-fed rats.

Furthermore, we found an increase in the abundance of Proteobacteria phylum followed by CAF. Interestingly, obese animals that received Zn had an even higher abundance of this phylum. It is suggested that the increase in Proteobacteria is associated with an imbalance of the gut microbiota. Hence, it is a potential marker for dysbiosis [66]. It has already been reported that the increased colonization of Proteobacteria is associated with a high-fat diet (HFD) in both human and rodent models [60,67,68].

Among the analyzed genera, we found a decrease in the abundance of *Lactobacillus* following CAF and Zn supplementation, but Zn diminished *Lactobacillus* only in lean animals. *Lactobacillus* are beneficial bacteria with health-promoting properties which have been reported as contributors to host metabolism [68,69]. *Akkermansia* is a member of the Verrucomicrobia phylum that corroborates the maintenance of metabolic homeostasis. It increases goblet cell density, stimulates mucin production, and improves intestinal barrier integrity [70,71]. Indeed, studies have described the health-promoting effects of *Akkermansia* on energy metabolism and metabolic functions such as insulin sensitivity, dyslipidemia, and even cognition [72,73,74]. Interestingly, at the same time that *Akkermansia* improves mucin production, it appears to be also involved in mucin degradation [66,75]. *Akkermansia* is highly responsive to diet change. Previous DIO studies reported a higher prevalence of this genus [73,76] or even a decrease in its abundance [73,77]. Here, we observed an increase in *Akkermansia* abundance following CAF. Thus, the mechanisms related to alterations in the *Akkermansia* population warrant further investigation. As for *Blautia* genera, we found an increase in its abundance following CAF. This finding is in accordance with previous studies on obesogenic diets both in humans and rodents [78,79]. Again, while some studies reported deleterious effects linked with a higher abundance of *Blautia* [80,81], others reported beneficial effects [82].

Concerning the diversity taxa, it is consistently described that higher diversity characterizes healthy gut microbiota [58,83]. Indeed, lower microbiota richness is associated with an obesogenic diet in both humans and animals [42,79]. We found a reduction in alpha diversity in CAF-fed rats, in accordance with other studies [42,44,84]. However, we did not find an effect of Zn supplementation in this analysis. Previous studies showed the divergent effects of Zn on microbiota diversity. While some demonstrated that Zn supplementation tends to decrease overall bacterial richness [85], others showed that low dietary Zn supplementation had no effect [86]. In summary, our results suggest that Zn supplementation impacts bacterial communities by supporting or restricting the growth of selected taxa, corroborating with previous studies [75,85]. However, CAF is composed of several industrialized foods, and our study does not provide evidence of the impact of a particular diet component on the microbiota, which is a limitation. Future studies should address this point.

SCFA are microbiota metabolites that modulate several host functions. Butyrate interferes in host metabolism and immunity by regulating satiety [87,88], exerting an anti-inflammatory role [32,89], maintaining epithelial integrity in the intestine [39], and also providing an energy source for colonocytes [89]. Our findings showed a decrease in butyrate levels in CAF-fed animals, with no effect of Zn supplementation. The lower concentration of butyrate is in accordance with the decreased abundance of the Firmicutes phylum since butyrate-producing species belong to this phylum [62,90].

Acetate represents the most abundant SCFA in the body, being found in higher concentrations in blood and peripheral tissues [88,91]. It is a metabolite synthesized by the phylum Bacteroidetes, and is essential for the growth of several bacteria [62]. Acetate is the main SCFA synthesized by intestinal bacteria and exerts an important effect on the body’s energy regulation [58,92]. Our findings demonstrated a significantly lower acetate concentration in the CAF group compared to the CAF + Zn group. Hence, Zn supplementation was capable of reestablishing the acetate concentration in CAF + Zn animals. Once again, a higher concentration of acetate is in accordance with the enhanced abundance of Bacteroidetes phylum observed in CAF + Zn rats. A previous pre-clinical study reported an increase in the presence of Gram-negative facultative anaerobic bacteria and the colonic concentration of SCFAs followed by Zn supplementation [93,94]. We also showed lower concentrations of isovalerate following CAF and an increase in valerate concentration in rats supplemented with Zn. A beneficial effect of valerate in intestinal epithelial integrity and an improvement in gastrointestinal function are reported [95]. Thus, Zn may exert a beneficial role by increasing valerate and acetate levels. 

Regarding the concentration of SFAs in the colon, we observed an increase in several MCFAs, LCFAs, and VLCFAs following the CAF. The undecanoic and myristoleic fatty acids showed an interaction effect between diet and Zn supplementation. It has already been established that Zn is involved in several biochemical and enzymatic processes. Additionally, it is a part of desaturases and elongases and appears to influence the fatty acid profile. Changes in the amount of saturated fatty acids have already been reported following Zn supplementation [96]. Overall, we expected a higher concentration of these fatty acids due to CAF composition, characterized by a high saturated fatty acid content. Our findings are in accordance with previous studies concerning the quality and amount of dietary fat that modulates the gut microbiota composition and impacts metabolic health [97,98]. Moreover, it has already been reported that a high intake of dietary saturated fatty acids contributes to the establishment of low-grade systemic inflammation and enhances the risk of developing obesity and cancer-related diseases, and even impacts the onset of Alzheimer’s and other dementias [78,99].

In addition, SFAs act as non-microbial agonists of TLR-4, sharing a common mechanism of action with lipopolysaccharide (LPS), a constituent of intestinal bacteria related to endotoxemia [100]. Moreover, it was demonstrated that saturated fatty acids stimulate the nuclear transcription factor kappa B (NF-κB) pathway in a TLR-4-dependent manner, leading to an increase in pro-inflammatory cytokine synthesis such as IL-6 and TNF-α [96,101]. Among the analyzed fatty acids in the present study, palmitate and stearate are the main representatives of saturated fatty acids in the human organism, and were both increased in CAF-fed rats [96].

The excessive consumption of SFAs has several consequences on the intestinal gut microbiota, including the dysbiosis and dysfunction of the gut barrier, enhancing the proinflammatory state [13,31]. Here, we analyzed the protein expression of ZO-1 and claudin-5 in the cerebral cortex, hippocampus, and intestine. There was no change in ZO-1 expression in these three regions. Mice that received an HFD for 14 weeks showed a decrease in the protein expression of claudin-5 and occludin in the frontal cortex, while ZO-1 was not affected by diet [102]. Regarding the claudin-5 protein expression in the hippocampus, we did not find differences between the groups. However, our findings showed a decrease in claudin-5 protein expression in the cerebral cortex of CAF-fed animals, with no effect of Zn supplementation. Intriguingly, we also observed an effect of diet in the claudin-5 expression in the intestine, which was increased in CAF-fed rats, with no effect of Zn supplementation. It has already been reported that the composition and distribution of epithelial claudins vary spatially along the length of the intestine. In intestinal inflammatory disorders, there is an overexpression of claudin-1, -2, and -18, simultaneously, with the downregulation of claudin-3, -4, -5, -7, -8, and -12. Such changes can modify epithelial barrier function and mucosal homeostasis [24,25]. Thus, we speculate that the damage caused by CAF may alter the expression of other proteins, such as occludins or other claudins, which were not investigated here. Claudin-5 may increase to compensate for other alterations in the tight junction complex. Although we found no effect of Zn supplementation on the expression of the tight junction proteins, it has already been described that Zn may have a protective effect on the intestinal barrier. The mechanisms are not fully elucidated, but zinc-mediated protection seems to be due to the stimulation of GPR39, a zinc-sensing receptor involved in barrier regulation [103]. Furthermore, Zn significantly enhanced the barrier function in an in vitro model [104].

The central nervous system is another important target of the harmful effects of obesity. It has already been described that there is a reciprocal interaction between gut microbiota and the central nervous system. This bidirectional communication, known as the gut–brain axis, is regulated via immune and neuroendocrine signals [105,106,107]. Several studies have reported the association between gut microbiota dysbiosis and neurologic disorders, such as cognitive impairments, Alzheimer’s disease [99], Parkinson’s disease [108], autism spectrum [109], and mood disorders [110]. Thus, we evaluated the effect of CAF on synaptophysin and BDNF as markers of neuroplasticity to further investigate the relationship between gut and brain in obesity. 

Although the mechanisms are not fully elucidated, it appears that Zn implicates in the modulation of neurotrophic signaling [111,112,113]. In this study, we did not find differences in BDNF expression in the hippocampus. However, studies in rodents reported that Zn supplementation could increase the expression of BDNF in the hippocampus and prevent cognitive impairment. Additionally, it seems that the effectiveness of Zn supplementation in increasing BDNF in obese mice was dose-dependent [114,115]. We have previously shown that Zn diminished neuroinflammation and improved memory in obese rats. Thus, this mechanism of neuroprotection may be related to the reduction of inflammation without affecting BDNF expression. On the other hand, Zn supplementation in obese individuals increased the serum concentration of BDNF [116]. However, studies in humans have a limitation due to the circulating BDNF not necessarily reflecting its availability and concentration in encephalic structures [117,118].

We also evaluated synaptophysin as a marker of neuroplasticity. Although changes in synaptophysin expression in the cerebral cortex were not seen, the CAF reduced its expression in the hippocampus. A decreased synaptophysin expression in the hippocampus of rodents with an obesity-induced cognitive deficit has already been reported [119]. Cai and collaborators demonstrated that hyperglycemia and hyperlipidemia induced by obesity might enhance the hippocampal endoplasmic reticulum stress and impair the expression of BDNF and synaptophysin, consequently leading to memory and learning dysfunction in rats [120]. However, it was previously shown that a low dose of Zn was efficient in increasing synaptophysin following a high-fat diet in mice [115].

## 5. Conclusions

In summary, we showed that the chronic consumption of the CAF causes dysbiosis, morphological change, and a decrease in SCFA levels in the colon, along with increased saturated fatty acids. The BBB may also be compromised in CAF-fed animals since claudin-5 expression is reduced in the cerebral cortex. Additionally, synaptophysin was decreased in the hippocampus, which might affect synaptic function. Zinc supplementation did not protect from dysbiosis. However, it increased acetate levels, which suggests a partial beneficial effect of Zn. In the brain, Zn did not show a neuroprotective role in the present study. Nevertheless, in accordance with previous studies, we have demonstrated a beneficial role of Zn in neuroinflammation and cognitive function in obesity. Thus, the consumption of ultra-processed foods, as provided by the CAF, causes severe obesity. In this condition, Zn might not be sufficient to protect or revert obesity-related dysfunctions such as dysbiosis.

## Figures and Tables

**Table 1:** Medium-chain fatty acid (MCFA), long-chain fatty acid (LCFA) and very long-chain fatty acid (VLCFA) concentrations in the proximal portion of the colon (µg/mg).

Fatty Acid	Classification	CT	CT + Zn	CAF	CAF + Zn	Two-Way ANOVA Results			
						Interaction	Diet Effect	Zn Effect	Bonferroni’s Post Hoc Test
**MCFA (6–12 carbons)**									
Caprylic	Saturated	9.36 ± 4.14	5.78 ± 1.00	3.36 ± 0.93	3.67 ± 0.90		0.0479		
Decanoic	Saturated	0.32 ± 0.03	0.23 ± 0.04	0.65 ± 0.17	0.51 ± 0.10	ns	0.0065	ns	ns
Octanoic	Saturated	0.11 ± 0.03	0.09 ± 0.03	0.29 ± 0.08	0.19 ± 0.09	ns	0.0326	ns	ns
Lauric	Saturated	1.86 ± 0.31	1.44 ± 0.36	8.207 ± 2.05	5.72 ± 2.54	ns	0.0025	ns	ns
Undecanoic	Saturated	19.35 ± 3.12	11.07 ± 2.25	16.99 ± 1.42	19.49 ± 1.62	0.0254	ns	ns	CT vs. CT + Zn (0.0285)
**LCFA (13–21 carbons)**									
Elaidic	Unsaturated trans fatty acid	61.41 ± 13.43	48.10 ± 15.02	244.9 ± 40.66	223.6 ± 44.51	ns	0.0001	ns	ns
Heptadecanoic	Saturated	3.71 ± 0.91	3.73 ± 0.99	3.82 ± 0.93	3.38 ± 1.24	ns	ns	ns	ns
Linoleic	Unsaturated	110.7 ± 18.36	76.81 ± 23.58	458.9 ± 114.2	322.6 ± 109.3	ns	0.0009	ns	ns
Myristic	Saturated	2.37 ± 0.85	2.50 ± 0.89	8.48 ± 0.89	5.77 ± 0.47	ns	0.0001	ns	ns
Palmitic	Saturated	28.83 ± 7.76	39.78 ± 9.52	47.52 ± 9.32	86.56 ± 27.04	ns	0.0049	ns	ns
Pentadecanoic	Saturated	14.21 ± 2.05	13.43 ± 1.07	18.61 ± 5.09	12.84 ± 1.22	ns	ns	ns	ns
Stearic	Saturated	59.69 ± 11.16	60.76 ± 10.34	34.22 ± 5.74	39.11 ± 4.99	ns	0.0179	ns	ns
Myristoleic	Unsaturated	29.96 ± 7.17	33.63 ± 7.39	52.10 ± 7.96	40.38 ± 10.15	0.0215	ns	ns	ns
Tridecanoic	Saturated	6.96 ± 1.52	4.98 ± 0.48	5.90 ± 1.03	5.65 ± 0.97	ns	ns	ns	ns
**VLCFA (≥22 carbons)**									
Behenic	Saturated	31.91 ± 7.10	32,14 ± 7.29	122.0 ± 33.77	182.6 ± 32.56	ns	0.0001	ns	ns
Tricosanoic	Saturated	25.90 ± 6.64	23.64 ± 3.88	43.57 ± 13.66	38.23 ± 13.53	ns	ns	ns	ns
Heneicosanoic	Saturated	11.31 ± 2.75	6.23 ± 2.05	27.86 ± 5.99	19.42 ± 4.10	ns	0.0012	ns	ns
Lignoceric	Saturated	12.68 ± 5.60	33.14 ± 7.72	19.64 ± 7.05	16.92 ± 5.35	ns	ns	ns	ns

Values are expressed as mean ± SEM. *p* values are described when significant differences were found. ns (non-significant); CT (control diet); CAF (cafeteria diet); Zn (zinc). *n* = 5/7 animals/group.

## Data Availability

The data that support the findings of this study are available on reasonable request from the corresponding author.
